# Multidrug-Resistant Tuberculosis in Europe, 2010–2011

**DOI:** 10.3201/eid2103.141343

**Published:** 2015-03

**Authors:** Gunar Günther, Frank van Leth, Sofia Alexandru, Neus Altet, Korkut Avsar, Didi Bang, Raisa Barbuta, Graham Bothamley, Ana Ciobanu, Valeriu Crudu, Manfred Davilovits, Martin Dedicoat, Raquel Duarte, Gina Gualano, Heinke Kunst, Wiel de Lange, Vaira Leimane, Cecile Magis-Escurra, Anne-Marie McLaughlin, Inge Muylle, Veronika Polcová, Emanuele Pontali, Christina Popa, Rudolf Rumetshofer, Alena Skrahina, Varvara Solodovnikova, Victor Spinu, Simon Tiberi, Piret Viiklepp, Christoph Lange, TBNET

**Affiliations:** University of Namibia School of Medicine, Windhoek, Namibia (G. Günther, C. Lange);; German Center for Infection Research, Research Center Borstel, Borstel, Germany (G. Günther, C. Lange);; University of Amsterdam, Amsterdam, the Netherlands (F. van Leth);; Institute of Phthisiopneumology, Chisinau, Moldova (S. Alexandru, A. Ciubanu, V. Crudu);; Hospital Universitari Vall d’Hebron, Barcelona, Spain (N. Altet);; Jordi Gol University, Barcelona (N. Altet);; Asklepios Klinik Gauting, Gauting, Germany (K. Avsar);; Statens Serum Institut, Copenhagen, Denmark (D. Bang);; Herlev Hospital, Herlev, Denmark (D. Bang);; Balti Municipal Hospital, Balti, Moldova (R. Barbuta);; Homerton University Hospital, London, UK (G. Bothamley);; National TB Reference Laboratory, Chisinau, Moldova (V. Crudu);; Tartu University Lung Hospital, Tartu, Estonia (M. Danilovits);; University of Warwick, Coventry, UK (M. Dedicoat);; Heart of England Foundation Trust, Birmingham, UK (M. Dedicoat, H. Kunst);; Vila Nova de Gaia/Espinho Medical School, Vila Nova de Gaia, Portugal (R. Duarte);; Porto University, Porto, Portugal (R. Duarte);; National Institute for Infectious Diseases L. Spallanzani, Rome, Italy (G. Gualano);; Queen Mary University, London (H. Kunst);; University Medical Center Groningen, Groningen, the Netherlands (W. de Lange);; Riga East University Hospital, Riga, Latvia (V. Leimane);; Radboud University Medical Centre, Nijmegen/Groesbeek, the Netherlands (C. Magis-Escurra);; St. James's Hospital, Dublin, Ireland (A.-M. McLaughlin);; University Medical Center St. Pieter, Brussels, Belgium (I. Muylle);; Thomayer University Hospital, Prague, Czech Republic (V. Polcavá);; Galliera Hospital, Genoa, Italy (E. Pontali);; Marius-Nasta-Institut, Bucharest, Romania (C. Popa, V. Spinu);; Otto Wagner Hospital, Vienna, Austria (R. Rumetshofer);; Republican Research and Practical Centre for Pulmonology and Tuberculosis, Minsk, Belarus (A. Skrahina, Varvara Solodovnikova);; Azienda Ospedaliera della Valtellina e della Valchiavenna E. Morelli Reference Hospital for MDR and HIV-TB, Sondalo, Italy (S. Tiberi);; Barts Health National Health Service Trust, London (S. Tiberi);; National Institute for Health Development, Tallinn, Estonia, (P. Viiklepp);; Karolinska Institute, Stockholm, Sweden (C. Lange)

**Keywords:** tuberculosis and other mycobacteria, multidrug-resistant tuberculosis, MDR TB, extensively drug-resistant tuberculosis, XDR TB, drug resistance, risk, Mycobacterium tuberculosis, bacteria, TBNET, second-line drugs, Europe

## Abstract

Drug-resistant *Mycobacterium tuberculosis* is challenging elimination of tuberculosis (TB). We evaluated risk factors for TB and levels of second-line drug resistance in *M. tuberculosis* in patients in Europe with multidrug-resistant (MDR) TB. A total of 380 patients with MDR TB and 376 patients with non–MDR TB were enrolled at 23 centers in 16 countries in Europe during 2010–2011. A total of 52.4% of MDR TB patients had never been treated for TB, which suggests primary transmission of MDR *M. tuberculosis*. At initiation of treatment for MDR TB, 59.7% of *M. tuberculosis* strains tested were resistant to pyrazinamide, 51.1% were resistant to ≥1 second-line drug, 26.6% were resistant to second-line injectable drugs, 17.6% were resistant to fluoroquinolones, and 6.8% were extensively drug resistant. Previous treatment for TB was the strongest risk factor for MDR TB. High levels of primary transmission and advanced resistance to second-line drugs characterize MDR TB cases in Europe.

## Abstract 2

Ongoing transmission, high levels of drug resistance, and poor diagnostic

Emergence of drug-resistant tuberculosis (TB) threatens the goal of TB elimination (*1*). Multidrug-resistant (MDR) TB is defined by in vitro resistance of *Mycobacterium tuberculosis* to at least both of the 2 most effective drugs for treatment (rifampin and isoniazid). Extensively drug-resistant TB (XDR TB) is defined as MDR TB plus in vitro resistance to at least 1 second-line injectable drug (amikacin, capreomycin, or kanamycin) plus resistance to any of the fluoroquinolones (e.g., ofloxacin, levofloxacin, or moxifloxacin) (*2*). In the World Health Organization (WHO) European Region, the estimated incidence of patients with MDR TB differs markedly: 1.6 cases/100,000 persons in the 29 European Union/European Economic Area countries and 16.8 cases/100,000 persons in the 24 other countries of the region in 2012 (Technical Appendix Table 1) (*3*). The actual number of patients with MDR TB living in this region may be much higher because a substantial proportion of patients are never screened for drug-resistant TB before starting treatment, partly because of a lack of diagnostic capacity (*3*).

**Table 1:** Baseline characteristics of patients with MDR TB in TBNET study in Europe, 2010–2011*

Characteristic	Incidence of TB			All sites
	Low†	Intermediate‡	High§	
Patients	103 (27.1)	86 (22.6)	191 (50.3)	380 (100)
Age, y	31 (27–39)	41 (26–49)	37 (28–50)	36 (27–47)
Body mass index	20 (19–23)	21 (18–22)	21 (18–23)	21 (19–23)
Male sex	50 (48.5)	56 (65.1)	133 (69.6)	239 (62.9)
Foreign born	88 (85.4)	5 (5.8)	1 (0.5)	94 (24.7)
Current smoker				
Yes	65 (63.1)	33 (38.4)	94 (49.2)	192 (50.5)
Unknown	9 (8.7)	2 (2.3)	1 (0.5)	13 (3.4)
HIV infected				
Yes	8 (7.8)	8 (9.3)	9 (4.7)	25 (6.6)
Not tested	8 (7.8)	0	1 (0.5)	9 (2.4)
Unknown	0	0	1 (0.5)	1 (0.3)
Diabetes				
Yes	3 (2.9)	4 (4.7)	9 (4.7)	16 (4.2)
Unknown	5 (4.9)	2 (2.3)	1 (0.5)	8 (2.1)
TB contact				
No	49 (47.6)	39 (45.3)	68 (35.6)	156 (41.1)
Yes, no MDR case	1 (1.0)	16 (18.6)	23 (12.0)	54 (14.2)
Yes, MDR case	15 (14.6)	2 (2.3)	5 (2.6)	8 (2.1)
Unknown	38 (36.9)	29 (33.7)	95 (49.7)	162 (42.6)
TB treatment				
Yes	42 (40.8)	23 (26.7)	116 (60.7)	181 (47.6)
Unknown	3 (2.9)	0	0	3 (0.8)
Classification of current TB episode				
New	61 (59.2)	64 (74.4)	74 (38.7)	199 (52.4)
Relapse	23 (22.3)	20 (23.3)	48 (25.1)	91 (23.9)
Treatment failure	8 (7.8)	2 (2.3)	37 (19.4)	47 (12.4)
Chronic	1 (1.0)	0	14 (7.3)	15 (3.9)
Returned defaulter	9 (8.7)	0	18 (9.4)	27 (7.1)
Unknown	1 (1.0)	0	0	1 (0.3)
Location of TB infection				
Pulmonary	74 (71.8)	79 (91.9)	191 (100.0)	344 (90.5)
Extrapulmonary	14 (13.6)	2 (2.3)	0	16 (4.2)
Pulmonary and extrapulmonary	15 (14.6)	5 (5.8)	0	20 (5.3)
Radiologic findings				
No pathologic changes	8 (7.8)	1 (1.2)	1 (0.5)	10 (2.6)
Cavitary	40 (38.8)	52 (60.5)	147 (77.0)	239 (62.9)
Noncavitary	52 (50.5)	33 (38.4)	43 (22.5)	128 (33.7)
Unknown	3 (2.9)	0	0	3 (0.8)
Bacteriologic result				
Smear +, culture +	64 (62.1)	39 (45.3)	142 (74.3)	245 (64.5)
Smear –, culture +	38 (36.9)	45 (52.3)	47 (24.6)	130 (34.2)
Unknown	1 (1.0)	0	0	1 (0.3)
Age, y				
<18	3 (2.9)	0	6 (3.1)	9 (2.4)
18–24	17 (16.5)	14 (16.3)	25 (13.1)	56 (14.7)
25–44	68 (66.0)	34 (39.5)	91 (47.6)	193 (50.8)
45–64	12 (11.7)	28 (32.6)	62 (32.5)	102 (26.8)
≥65	3 (2.9)	2 (2.3)	2 (1.0)	7 (1.8)
Unknown	0	8 (9.3)	5 (2.6)	13 (3.4)
Currently employed				
Yes	46 (44.7)	35 (40.7)	63 (33.3)	144 (37.9)
Unknown	7 (6.8)	6 (7.0)	1 (0.5)	14 (3.7)
Work in a high-risk setting for TB				
Yes	8 (7.8)	6 (7.0)	9 (4.7)	23 (6.1)
Unknown	16 (15.5)	12 (14.0)	107 (56.0)	135 (35.5)
Excessive alcohol consumption				
Yes	11 (10.7)	20 (23.3)	48 (25.1)	79 (20.8)
Unknown	10 (9.7)	1 (1.2)	1 (0.5)	12 (3.2)
Imprisonment before current diagnosis				
Yes	4 (3.9)	8 (9.3)	18 (9.4)	30 (7.9)
Unknown	12 (11.7)	1 (1.2)	1 (0.5)	14 (3.7)
Current homelessness				
Yes	9 (8.7)	2 (2.3)	5 (2.6)	16 (4.2)
Unknown	2 (1.9)	2 (2.3)	1 (0.5)	5 (1.3)
Injectable drug use				
Yes	7 (6.8)	7 (8.1)	10 (5.2)	24 (6.3)
Unknown	12 (11.7)	2 (2.3)	15 (7.9)	29 (7.6)
Hepatitis B				
Yes	14 (13.6)	2 (2.3)	1 (0.5)	17 (4.5)
Unknown	20 (19.4)	5 (5.8)	78 (40.8)	103 (27.1)
Hepatitis C				
Yes	12 (11.7)	5 (5.8)	2 (1.0)	19 (5.0)
Unknown	22 (21.4)	22 (25.6)	81 (42.4)	125 (32.9)
Use of TNF inhibitor				
Yes	1 (1.0)	0	4 (2.1)	5 (1.3)
Unknown	7 (6.8)	5 (5.8)	42 (22.0)	54 (14.2)
Silicosis				
Yes	0	1 (1.2)	0	1 (0.3)
Unknown	3 (2.9)	1 (1.2)	1 (1.1)	5 (1.3)
Vaccination with *Mycobacterium bovis* BCG				
Yes	42 (40.8)	72 (83.7)	184 (96.3)	300 (78.5)
Unknown	58 (56.3)	14 (16.3)	6 (3.1)	78 (20.4)

*Values are no. (%) or median (interquartile range). Unweighted analysis was used. MDR TB, multidrug-resistant tuberculosis; +, positive; –, negative;TNF, tumor necrosis factor. †Austria, Belgium, Czech Republic, Denmark, Germany, Great Britain, Ireland, Netherlands, Italy, and Spain. ‡Belarus, Estonia, Latvia, and Portugal. §Moldova and Romania.

MDR TB is associated with poor treatment outcomes (*1**,**2**,**4*). The proportion of treatment success in patients with MDR TB was only 54% in an individual patient data metaanalysis of >9,000 patients from 32 observational studies (*5*). Results from this cohort showed that additional resistance to fluoroquinolones in patients with MDR TB reduced treatment success to 48%; patients with XDR TB were treated successfully in 40% of cases (*6*), which approached treatment outcomes similar to those of the pre–antimicrobial drug era (*4*). A recent surveillance report from the EU reported 32.2% treatment success for MDR TB and 19.1% treatment success for XDR TB (*7*).

Detailed information about characteristics, management, and outcomes of patients with MDR TB in Europe is scarce but essential to inform health policy makers and optimize disease management (*8*). We compared baseline characteristics and risk factors for patients with MDR TB, as well as availability and results of drug susceptibility testing (DST) for second-line drugs for treatment of TB, in a cohort of patients from 16 countries in Europe with low, intermediate, and high incidence of TB, who had started first-line or second-line TB treatment.

## Methods

### Participating Sites

TBNET is a European consortium for clinical research in the field of TB (*9*). This study was conducted at 23 TBNET sites in 16 countries in Europe: 2 with a high (>100 cases/100,000 persons) incidence of TB, 4 with an intermediate (20–100 cases/100,000 persons) incidence, and 10 with a low (<20 cases/100,000 persons) incidence (Figure). Stratification was based on WHO/European Centre for Disease Prevention and Control estimates of TB incidence during the study period (2010–2011) (*10*). The number of study participants per country is shown in Technical Appendix Table 1.

**Figure:**
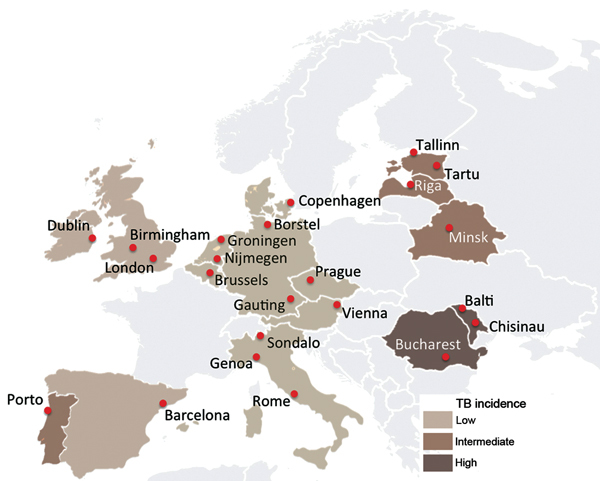
TBNET study sites in the Pan European network for study and clinical management of drug-resistant tuberculosis (TBPAN-NET) project. Stratification is based on the incidence of tuberculosis (TB) reported during 2010–2011, which matched the inclusion period of the study. Data for 2011 were obtained from the European Centre for Disease Control and Prevention (*10*). Low TB incidence, <20 cases/100,000 persons; intermediate TB incidence, 20–100 cases/100,000 persons; high TB incidence, >100 cases/100,000 persons.

### Study Population

After informed consent was obtained, patients starting treatment for a new episode of culture-confirmed TB with resistance to at least rifampin and isoniazid (MDR TB cohort) were eligible for enrollment. Patients were included prospectively by using consecutive inclusion during January 2010–December 2011 at each site. In Belarus, Latvia, Moldova, and Romania, additional enrollment was stopped when a preagreed number of patients were enrolled to avoid overrepresentation of patients from these countries in the cohort.

For each MDR TB patient, 1 patient with non–MDR TB (pan drug–susceptible, monoresistant, or polydrug-resistant TB [*11*]) was enrolled at each center at the time of enrollment of the MDR TB patient: these additional patients were denoted as controls. Controls were selected on the basis of DST results that identified non–MDR TB, and that were obtained at the closest date before enrollment of an MDR TB patient at the same site.

Because of this selection process, a limited number of patients (41, 5.4%) started treatment before the study began in January 2010, but none started treatment before January 2007. However, we maintained consecutive inclusion for MDR TB patients. This feature ensured an acceptable sample size for countries with a low incidence of TB during the inclusion period.

### Data Collection

Data collection used an electronic case record form designed in Open Clinica (http://www.openclinica.com). A paper version of this form was used in Moldova, Romania, Estonia, and Belarus, where internet access was not always available. All investigators were initially trained onsite, and continuous training was ensured through annual investigator meetings, regular site visits, and newsletters.

### Laboratory Testing

Routine data were obtained from local laboratory reports for sputum smear microscopy, sputum culture, and DST for first-line and second-line drugs and, when available, *M. tuberculosis*–specific nucleic acid amplification tests. All laboratories at study sites were subjected to quality control through the WHO Supranational Reference Laboratory Network.

### Study Outcome

We analyzed characteristics of the cohort at the time of enrollment. We also assessed factors associated with MDR TB in a cross-sectional approach.

### Data Management

Data management included regular data checks on key variables for missing data and inconsistencies. The study coordinator, a study monitor, and a trained study nurse performed routinely manual plausibility checks and clarified inconsistencies with the investigators.

### Statistical Analysis

Descriptive statistics are reported as frequencies or medians, where appropriate. Risk factor analysis was performed by using univariable and multivariable logistic regression. We used robust SEs to adjust for clustering by country. All variables with <20% missing data were assessed for inclusion in the models. Missing data for included variables were coded as separate categories to prevent listwise deletion of observations. Age was dichotomized at 45 years to align with values in a previous study (*12*). The variables age and sex were purposefully included in the multivariable analysis in which other variables were included on the basis of the Wald statistic (<0.1) and the change in the pseudo R^2^ (>10%) because a robust SE precludes formal use of the log-likelihood ratio test. In a sensitivity analysis, we repeated multivariable logistic regression with the inclusion of a sampling weight for the MDR TB patients (inverse of the sampling fraction with expected number of MDR TB patients in the country as denominator) (Technical Appendix Table 5). Non–MDR TB patients were given a weight of 1. The weighted analyses assessed the potential effect of unbalanced contribution of countries in the cohort. Goodness-of-fit was assessed by using the F-adjusted mean residual test.

Drug resistance was expressed as the proportion of isolates tested and the proportion of isolates that were resistant. Corresponding frequencies when applying sampling weights and analysis by a complex survey approach (*13**,**14*) are shown in Technical Appendix Table 4.

### Ethics

Patient information and consent forms were translated into local languages when needed. The study was approved by the Ethics Committee of the University of Lübeck (Lübeck, Germany). The study protocol was approved by the local ethics committee at all participating centers. Written informed consent was obtained from all patients according to site-specific regulations. Data were collected pseudonymously and stored on a secured server.

## Results

### Cohort Characteristics

The cohort consisted of 380 MDR TB patients and 376 non–MDR TB controls. Descriptive characteristics of the MDR TB cohort are shown in Table 1 and those for the non–MDR TB cohort in Technical Appendix Table 2. Both groups had predominantly male patients. The median age was 36 years (interquartile range 27–47 years) for the MDR TB patients and 41 years (interquartile range 31–54 years) for the controls. The proportion of foreign-born patients with MDR TB in countries of low, intermediate, and high TB incidence was 85.4%, 5.8% and 0.5%, respectively. Similar proportions were observed in controls (56.3%, 5.7% and 2.1%, respectively). Of 94 foreign-born patients, 60 (64%) were from countries of the European region of WHO, 17 (18%) from Russia, 18 (19%) from Southeast Asia, 11 (12%) from sub-Saharan Africa, 1 (1%) from North Africa, and 4 (4%) from South America. Smoking was common in both groups (50.5% for MDR TB patients and 41.5% for controls).

**Table 2:** Drug resistance profiles for first-line and second-line drugs used for treatment of multidrug-resistant tuberculosis in TBNET study in Europe, 2010–2011*

Drug†	Incidence of TB in region								All MDR TB patients, n = 380	
	Low, n = 103†			Intermediate n = 86‡			High n = 191§			
	Tested	Resistant		Tested	Resistant		Tested	Resistant	Tested	Resistant
First-line										
Pyrazinamide	97 (94.2)	52 (53.6)		70 (81.4)	49 (71.0)		10 (5.2)	4 (40.0)	177 (45.0)	105 (59.7)
Ethambutol	99 (96.1)	50 (50.5)		85 (98.9)	55 (64.7)		187 (97.9)	115 (61.5)	371 (97.6)	220 (59.3)
Streptomycin	93 (90.3)	78 (83.9)		85 (98.9)	82 (96.5)		187 (97.9)	171 (91.4)	365 (96.1)	331 (90.7)
≥1 non–first line	101 (97.1)	66 (65.4)		86 (100)	64 (74.4)		173 (86.4)	64 (37.0)	360 (94.7)	194 (51.1)
Class II										
Amikacin	95 (92.2)	17 (17.9)		85 (98.8)	25 (29.4)		1 (0.5)	0	181 (47.6)	42 (23.2)
Kanamycin	39 (37.9)	8 (20.5)		79 (91.9)	37 (46.8)		170 (89.0)	23 (13.5)	288 (75.8)	68 (23.6)
Capreomycin	88 (85.4)	15 (17.0)		84 (97.7)	26 (31.0)		94 (49.2)	4 (4.3)	266 (71.1)	45 (16.9)
≥1 second-line inj.	100 (97.1)	24 (24.0)		86 (100)	42 (48.8)		170 (89.0)	27 (15.9)	356 (93.7)	93 (26.1)
Class III										
Ofloxacin	69 (67.0)	16 (23.2)		86 (100)	26 (30.2)		169 (88.5)	14 (8.3)	324 (85.3)	56 (17.3)
Levofloxacin	16 (15.5)	1 (6.2)		7 (8.1)	1 (14.3)		10 (5.2)	2 (20.0)	32 (8.4)	4 (12.5)
Moxiflocacin	61 (59.2)	14 (23.0)		12 (14.0)	3 (25.0)		0		73 (19.2)	17 (23.3)
≥1 fluoroquinolone	96 (96.2)	21 (21.9)		86 (100)	26 (30.2)		170 (89.0)	15 (8.8)	352 (92.6)	62 (17.6)
Class IV										
ETO/PTO	98 (95.1)	47 (48.0)		86 (100)	36 (41.9)		170 (89.0)	36 (21.2)	354 (93.2)	119 (31.3)
PAS	54 (52.4)	10 (18.5)		68 (79.1)	10 (14.7)		175 (91.6)	2 (1.1)	295 (77.6)	22 (7.5)
DCS/TRD	53 (51.5)	6 (11.3)		69 (80.2)	13 (18.8)		100 (52.4)	5 (5.0)	220 (57.9)	23 (10.6)
Class V										
Linezolid	62 (60.2)	2 (3.2)		6 (7.0)	0		1 (0.5)	0	69 (18.2)	2 (2.9)
Imipenem	0	0		0	0		0	0	0	0
Meropenem	1 (1.0)	1 (100)		0	0		0	0	1 (0.3)	1 (100)
AMX/CLV	0	0		0	0		0	0	0	0
Clarithromycin	17 (16.5)	3 (17.7)		0	0		0	0	17 (4.5)	3 (17.6)

*Values are no. (%) samples. Unweighted analysis was used. TB, tuberculosis; MDR TB, multidrug-resistant tuberculosis; inj, injectable; ETO/PTO, ethionamide/prothionamide; PAS, para-aminosalicylic acid; DCS/TRD, cycloserine/terizidone; AMX/CLV, amoxicillin/clavulanic acid. †Austria, Belgium, Czech Republic, Denmark, Germany, Great Britain, Ireland, Netherlands, Italy, and Spain. ‡Belarus, Estonia, Latvia, and Portugal. §Moldova and Romania.

HIV infection and diabetes mellitus were infrequently observed: 6.6% in MDR TB patients and 4.3% in controls for HIV, and 4.2% in MDR TB patients and 5.3% in controls for diabetes mellitus. The percentage of patients with MDR TB whose episode of active TB was their first was 52.4% (59.2%, 74.4%, and 38.7% in countries with low, intermediate, and high TB incidence, respectively).

### Drug Resistance Profiles

Among 380 patients with MDR TB, second-line *M. tuberculosis* DST profiles were available for 356 patients. Reasons for unavailable baseline DST results were 1) an initial diagnosis of MDR TB at a peripheral hospital and subsequent patient transfer to a central hospital where *M. tuberculosis* could not be grown in culture (n = 6); 2) contamination of cultures (n = 12); 3) insufficient growth in cultures (n = 4); 4) patient death between the first and second cultures (n = 1), and 5) unknown reason (n = 1). Among patients with MDR TB, 6.8% of cases fulfilled the definition of XDR TB. Drug resistance profiles for first-line and second-line drugs other than rifampin and isoniazid are shown in Table 2 for the MDR TB cohort, in Technical Appendix Table 3 for the MDR TB cohort compared with the non–MDR TB cohort, and in Technical Appendix Table 4 for the MDR TB cohort by weighted analysis.

**Table 3:** Risk factors for multidrug-resistant tuberculosis in patients in TBNET study in Europe, 2010–2011*

Factor	Non–MDR TB, n = 376	MDR TB, n = 380	Univariable analyisis		Multivariable analysis	
			OR (95% CI)	p value	OR (95% CI)	p value
Sex						
F	111	141	1	NA	1	NA
M	265	239	0.71 (0.52–0.97)	0.031	0.78 (0.53–1.14)	0.195
Age, y						
<45	212	258	1.73 (1.16–2.58)	0.007	1.90 (1.23–2.93)	0.004
≥45	155	109	1	NA	1	NA
Unknown	4	10	NA	NA	NA	NA
Body mass index						
<18	31	48	1.64 (0.94–2.85)	0.082	NA	NA
18–<25	276	261	1	NA	NA	NA
≥25	38	49	1.36 (0.65–2.87)	0.414	NA	NA
Currently employed						
Yes	144	144	1	NA	NA	NA
No	211	222	1.03 (0.71–1.49)	0.886	NA	NA
Unknown	16	14	NA	NA	NA	NA
Foreign born						
Yes	63	94	1.63 (1.12–2.38)	0.011	1.52 (0.89–2.61)	0.120
No	313	286	1	NA	1	NA
Imprisonment before current diagnosis						
Yes	15	30	2.05 (0.75–5.66)	0.164	1.27 (0.82–1.97)	0.280
No	345	336	1	NA	1	NA
Unknown	16	14	NA	NA	NA	NA
Current homelessness						
Yes	21	16	0.73 (0.43–1.24)	0.248	NA	NA
No	346	359	1	NA	NA	NA
Unknown	9	5	NA	NA	NA	NA
Injectable drug user						
Yes	13	24	1.87 (0.92–3.83)	0.084	1.32 (0.54–3.21)	0.541
No	332	327	1	NA	1	NA
Unknown	31	29	NA	NA	NA	NA
HIV infected						
Yes	16	25	1.57 (0.86–2.87)	0.146	1.78 (0.81–3.89)	0.151
No	320	345	1	NA	1	NA
Not tested	36	9	NA	NA	NA	NA
Unknown	4	1	NA	NA	NA	NA
Diabetes						
Yes	20	16	0.80 (0.32–1.98)	0.622	NA	NA
No	354	356	1	NA	NA	NA
Unknown	2	8	NA	NA	NA	NA
Previous TB treatment						
Yes	33	133	9.49 (7.05–12.76)	<0.001	10.71 (7.33–15.63)	<0.001
No	339	244	1	NA	1	NA
Unknown	4	3	NA	NA	NA	NA

*MDR TB, multidrug-resistant tuberculosis; OR, odds ratio; NA, not applicable.

DST for pyrazinamide and ethambutol was performed for 45.0% (177/380) and 97.6% (371/380) of strains from MDR TB patients and controls, respectively. Testing was performed for 94.7% (360/380) of strains for resistance to any second-line drug, 93.7% (356/380) for any second-line injectable drug, 92.6% (352/380) for any fluoroquinolone, and 93.2% (356/380) for ethionamide/prothionamide. Strains from MDR TB patients showed additional resistance to pyrazinamide (59.7%, 105/177), ethambutol (59.3%, 220/371), ≥1 second-line injectable drug (26.1%, 93/356), ≥1 fluoroquinolone (17.6%, 62/352), and ethionamide/prothionamide (31.3%, 119/354) (Table 2). The weighted analysis showed higher proportions of resistance to all drugs, except capreomycin, moxifloxacin, and ethionamide/prothionamide (Technical Appendix Table 4).

### Risk Factors for MDR TB

Risk factors for TB were compared between patients with MDR TB and controls. Previous treatment for TB (odds ratio 10.7, 95% CI 7.3–15.6) and age <45 years (OR 1.90, 95% CI 1.23–2.93) were identified as independent risk factors for MDR TB by multivariable analysis (Table 3). There was also a moderate association for sex and current homelessness with MDR TB by weighted analysis (Technical Appendix Table 5). No association was found between MDR TB and abnormal body mass index (<18 or >25), employment status, birth in a foreign country, history of imprisonment, injectable drug use, co-infection with HIV, or diabetes. The role of TB contact was not evaluated because data were not sufficiently robust because of a high percentage of unknown/unreliable results for self-reporting. Weighted analyses showed similar results with only minor differences in effect size.

## Discussion

We studied a multicenter cohort of patients with MDR TB at 23 referral centers across Europe and found high rates of drug resistance to second-line drugs for treatment of TB in circulating *M. tuberculosis* strains, and limited availability of second-line drug resistance testing in several countries with a high incidence of TB. Furthermore, we found evidence of ongoing transmission of MDR strains of *M. tuberculosis* in eastern Europe: 52.4% of patients with MDR TB were experiencing their first episode of TB. In countries in western Europe with a low incidence of TB, MDR TB is predominantly a disease of immigrants (*15*), which reflects the epidemiology of MDR TB in the country of origin. Only a few (8.9%) MDR TB patients were born outside the European region of WHO. Thus, interventions for the control of MDR TB should be specific for countries with high incidence of MDR TB, especially in eastern Europe (*16*).

Mathematical and epidemiologic models indicate that early diagnosis, effective treatment, and improved access to laboratory infrastructure could have a strong effect on the incidence of MDR TB in high-prevalence regions (*17*). However, few of such programmatic requirements are met at many sites in Europe at the present time (*18*).

Possible active transmission of strains causing MDR TB, as reflected by the large proportion of patients never having received TB treatment before in this European cohort, is consistent with recently reported data and deserves attention. A drug resistance survey conducted in Belarus in 2011 showed that 32.3% of new TB infections and 75.6% of previously treated TB infections had an MDR strain of *M. tuberculosis* (*19*). In Moldova, for which adequate surveillance data are available, 23.7% of new TB cases involve an MDR strain (*3*). A recent report of surveillance data in countries with >700 estimated MDR TB cases per year indicated that more than half of the reported pulmonary MDR TB cases were new cases (*20*).

More than 90% of strains from MDR TB patients had undergone DST for ≥1 second-line injectable drug and fluoroquinolone. The role of ethambutol and pyrazinamide for treatment of MDR TB is unclear. In our cohort, 97.6% and 45.0% of MDR TB strains were tested for resistance to ethambutol and pyrazinamide, respectively. In countries with a high incidence of TB, only 5.2% of MDR TB cases were tested for pyrazinamide resistance because of limited availability of liquid culture methods and special pH media requirements for pyrazinamide DST. Less than half of the strains tested were susceptible to these drugs. Currently, the mechanism of action of pyrazinamide in combination therapy and the relevance of in vitro DST for pyrazinamide are uncertain. Findings from this study raise questions about a universal recommendation to treat MDR TB with pyrazinamide throughout the entire course of treatment (*21*).

In our study cohort, 1 of 3 *M. tuberculosis* strains with resistance to at least rifampin and isoniazid were also resistant to protionamide/ethionamide, 1 of 4 were resistant to any second-line injectable drug, and 1 of 5 were resistant to a fluoroquinolone. Of all MDR TB cases, 6.8% fulfilled the definition of XDR TB. Surveillance data from the European Centre for Disease Prevention and Control indicated that 9.1% of cases of XDR TB in patients with MDR TB underwent second-line DST. Given the high proportion of strains that received a second-line DST, it is unlikely that these percentages are overstated because of preferred testing of patients at high risk for acquiring TB.

Our results are consistent with those from the Preserving Effective TB Treatment Study (PETTS) (*22*), which investigated second-line drug resistance in strains from 1,278 patients in 8 countries, including Latvia and Estonia, which were countries with study sites in this cohort. The main difference between PETTS study and ours was a high frequency of *M. tuberculosis* resistance to prothionamide/ethionamide in our cohort, which reflected the relatively higher frequency of treatment with this drug combination in eastern Europe than in other parts of the world (*23*). Recently published data from the PETTS study showed an increased risk of acquiring resistance to second-line drugs during treatment and increased baseline resistance (*24*). Increased resistance to second-line drugs is associated with higher proportions of treatment failures (*6*). It can be assumed, if one considers the findings from the PETTS study, that many of the patients in our cohort are at high risk for treatment failure.

Of particular concern is resistance to fluoroquinolones because these drugs are the core of new treatment regimens (*25*,*26*), including regimens for patients with drug-susceptible strains of *M. tuberculosis* (*26*). In our study, the capacity to perform DST for later-generation fluoroquinolones (levofloxacin and moxifloxacin) was only present for 19.2% of strains for levofloxacin and 8.4% of strains for moxifloxacin. Later generations of fluoroquinolones may still be effective for treatment of MDR TB in some patients when drug resistance to ofloxacin is documented (*27*). The capacity to perform DST for later generations of fluoroquinolones needs to be improved in the region.

Multivariable analysis showed that previous TB treatment and patient age <45 years showed an association with MDR TB (male sex and current homelessness showed an association in a weighted model). However, none of the other traditional risk factors for drug-resistant TB, such as HIV infection or birth in a foreign country (*12*), showed this association. Although previous treatment for TB and contact with persons infected with drug-resistant strains have been reported as strong risk factors for MDR TB, the role of HIV infection, young age, sex, and previous imprisonment are less clear (*12*,*28*). The high proportion of new cases and the lack of association of other traditional risk factors with drug-resistant TB suggest an increased role of ongoing transmission in the community outside established risk groups for becoming infected with drug-resistant strains of *M. tuberculosis* (*19**,**20**,**29*).

Our study has several major limitations. First, baseline data were obtained from an observational cohort study and were not derived from routine surveillance. Only 14 of 28 countries from the European Union and 2 countries outside the European Union were represented in the study. Site selection was based on voluntary participation in the study and being a center for the management of MDR TB. Because a high number of patients in Europe are being treated outside such centers, the generalizability of data might be limited. However, the included centers adhered to national policies regarding diagnosis and treatment of MDR TB patients and therefore reflect current practice. To provide a better estimation of representativeness of data for the situation in Europe, we additionally performed weighted analyses based on the sampling fraction and the expected number of reported MDR TB patients in the countries from which patients were recruited (online Technical Appendix). Results suggest that frequencies of drug resistance to second-line drugs might be underestimated by our analysis.

Second, some data collected were self-reported by patients and are prone to information bias. This limitation particularly applies to information on previous TB treatment in foreign-born patients, who might fear stigmatization in the country where treatment was provided.

Third, DST was performed at laboratories that used external quality control practices. However, quality control for testing of second-line drugs varies among sites and respective laboratories (*30*). Incompleteness of DST data for second-line drugs demonstrates the situation with which clinicians are confronted in making their management decisions and shows the need for scale up in laboratory testing, even in MDR TB reference centers in Europe.

Despite these limitations, our study identified 3 major concerns regarding TB in Europe. First, transmission of MDR strains of *M. tuberculosis* is ongoing. Second, diagnostic capacity is poor, especially for DST. Third, levels of resistance to second-line TB drugs are high. These factors must be addressed in any TB surveillance and control programs that are implemented.

**Technical Appendix.** Additional information for TBNET study of multidrug-resistant tuberculosis in Europe, 2010–2011.
