# Supplementary material for: Multidrug-Resistant Tuberculosis in Europe, 2010–2011
Source: Emerg Infect Dis. 2015 Mar;21(3):409–16. doi: 10.3201/eid2103.141343 (PMC4344280; doi:10.3201/eid2103.141343)
Supplement: Supplementary file 1 — Technical Appendix. Additional information for TBNET study of multidrug-resistant tuberculosis in Europe, 2010–2011. [file 14-1343-Techapp-s1.pdf]

Article DOI: http://dx.doi.org/10.3201/eid2103.141343

Multidrug-Resistant Tuberculosis in Europe, 2010–2011

Technical Appendix

Additional information for TBNET study of multidrug-resistant tuberculosis in Europe, 2010–2011

Technical Appendix Table 1. Epidemiology of drug-resistant tuberculosis in TBNET study in Europe, 2010–2011*

TB incidence and country	Estimated TB incidence in 2012, no. cases/100,000 persons†	No. reported TB cases in 2012†	Estimated MDR TB (%) among reported new TB cases in 2012†	Reported MDR TB cases among laboratory-confirmed TB cases in 2012†	No. MDR TB cases in cohort	No. non–MDR TB cases (controls) in cohort
Low						
Austria	7.9	648	6.9	27	12	12
Belgium	9.7	987	1.3	20	8	8
Czech Republic	5.3	605	1.5	4	5	4
Denmark	7.4	389	1.2	1	8	8
Germany	5.6	4,220	1.5	60	17	13
Ireland	8.6	325	1.1	5	4	5
Italy	6.7	3,142	3.9	74	20	18
The Netherlands	6.3	958	1.6	11	13	12
Spain	14.0	5,991	0.2	37	8	8
United Kingdom	15.0	8,751	1.3	81	8	8
Intermediate						
Belarus‡	70.0	5,118	35.0	1,564	31	32
Estonia	23.0	290	20.0	61	13	13
Latvia	53.0	993	11.0	106	31	31
Portugal	26.0	2,590	1.5	17	11	11
High						
Moldova‡	160.8	5,348	23.0	894	125	125
Romania§	96.0	18,197	2.8	530	66	68
Mean	32.2	3,660	6.9	218	NA	NA
Total	NA	NA	NA	NA	380	376

*MDR TB, multidrug-resistant tuberculosis; NA, not applicable.
†Source: Tuberculosis surveillance and monitoring in Europe 2014 (European Centre for Disease Prevention and Control/World Health Organization).
‡Countries outside the European Union.
§Romania had an estimated TB incidence of 102.6 cases/100.000 persons in 2011 (*1*). Therefore, Romania was classified as a high incidence country during the study.

Technical Appendix Table 2. Baseline characteristics of MDR TB and non–MDR TB cohort in TBNET study in Europe, 2010–2011*

	Incidence of TB							
	Low†		Intermediate‡		High§		All sites	
Characteristic	Non–MDR TB	MDR TB	Non–MDR TB	MDR TB	Non–MDR TB	MDR TB	Non–MDR TB	MDR TB
Patients	96 (25.6)	103 (27.1)	87 (23.1)	86 (22.6)	193 (51.3)	191 (50.3)	376 (100)	380 (100)
Age, y	37 (29– 57)	31 (27–39)	46 (34–54)	41 (26–49)	41 (31–53)	37 (28–50)	41 (31–54)	36 (27–47)
Body mass index	20 (19–22)	20 (19–23)	21 (19–23)	21.0 (18–22)	21 (19–22)	21 (18–23)	21 (19–22)	21 (19–23)
Sex								
M	58 (60.4)	50 (48.5)	60 (69.0)	56 (65.1)	147 (76.2)	133 (69.6)	265 (70.5)	239 (62.9)
Unknown	0	0	0	0	0	0	0	0
Foreign born								
Yes	54 (56.3)	88 (85.4)	5 (5.7)	5 (5.8)	4 (2.1)	1 (0.5)	63 (16.8)	94 (24.7)
Unknown	0	0	0	0	0	0	0	0
Current smoker								
Yes	54 (56.3)	65 (63.1)	26 (29.9)	33 (38.4)	76 (39.4)	94 (49.2)	156 (41.5)	192 (50.5)
Unknown	5 (5.2)	9 (8.7)	1 (1.1)	2 (2.3)	4 (2.1)	1 (0.5)	10 (2.7)	13 (3.4)
HIV infected								
Yes	2 (2.1)	8 (7.8)	6 (7.1)	8 (9.3)	8 (4.2)	9 (4.7)	16 (4.3)	25 (6.6)
Not tested	5 (5.3)	8 (7.8)	1 (1.2)	0	30 (15.7)	1 (0.5)	36 (9.7)	9 (2.4)
Unknown	1 (1.0)	0	2 (2.3)	0	2 (1.0)	1 (0.5)	4 (1.1)	1 (0.3)
Diabetes								
Yes	7 (7.3)	3 (2.9)	6 (6.9)	4 (4.7)	7 (3.6)	9 (4.7)	20 (5.3)	16 (4.2)
Unknown	0	5 (4.9)	0	2 (2.3)	2 (1.0)	1 (0.5)	2 (0.5)	8 (2.1)
TB contact								
No	52 (54.2)	49 (47.6)	55 (63.2)	39 (45.3)	130 (67.4)	68 (35.6)	237 (63.0)	156 (41.1)
Yes, non–MDR case	11 (11.5)	1 (1.0)	0	16 (18.6)	3 (1.6)	23 (12.0)	3 (0.8)	54 (14.2)
Yes, MDR case	0	15 (14.6)	13(14.9)	2 (2.3)	11 (5.7)	5 (2.6)	35 (9.3)	8 (2.1)
Unknown	33 (34.4)	38 (36.9)	19 (21.8)	29 (33.7)	49 (25.4)	95 (49.7)	101 (26.9)	162 (42.6)
History of TB Treatment								
Yes	9 (9.4)	42 (40.8)	2 (2.3)	23 (26.7)	22 (11.4)	116 (60.7)	33 (8.8)	181 (47.6)
Unknown	2 (2.1)	3 (2.9)	1 (1.1)	0	1 (0.5)	0	4 (1.1)	3 (0.8)
Classification current TB episode								
New	87 (90.6)	61 (59.2)	84 (96.6)	64 (74.4)	171 (88.6)	74 (38.7)	342 (91.0)	199 (52.4)
Relapse	7 (7.3)	23 (22.3)	2 (2.3)	20 (23.3)	19 (9.8)	48 (25.1)	28 (7.4)	91 (23.9)
Failure	0	8 (7.8)	0	2 (2.3)	1 (0.5)	37 (19.4)	1 (0.3)	47 (12.4)
Chronic	1 (1.0)	1 (1.0)	0	0	0	14 (7.3)	1 (0.3)	15 (3.9)
Returned defaulter	0	9 (8.7)	0	0	2 (1.0)	18 (9.4)	2 (0.5)	27 (7.1)
Unknown	1 (1.0)	1 (1.0)	1 (1.1)	0	0	0	2 (0.5)	1 (0.3)
Location of TB infection								
Pulmonary	78 (81.2)	74 (71.8)	79 (90.8)	79 (91.9)	192 (99.5)	191 (100.0)	349 (92.8)	344 (90.5)
Extrapulmonary	8 (8.3)	14 (13.6)	0	2 (2.3)	1 (0.5)	0	9 (2.4)	16 (4.2)
Pulmonary and extrapulmonary	9 (9.4)	15 (14.6)	8 (9.2)	5 (5.8)	0	0	17 (4.5)	20 (5.3)
Unknown	1 (1.0)	0	0	0	0	0	1 (0.3)	0
Radiologic findings								
No pathologic changes	9 (9.4)	8 (7.8)	0	1 (1.2)	11 (5.7)	1 (0.5)	20 (5.3)	10 (2.6)
Cavitary	36 (37.5)	40 (38.8)	51 (58.6)	52 (60.5)	120 (62.2)	147 (77.0)	207 (55.1)	239 (62.9)
Noncavitary	51 (53.1)	52 (50.5)	36 (41.4)	33 (38.4)	61 (31.6)	43 (22.5)	148 (39.4)	128 (33.7)
Unknown	0	3 (2.9)	0	0	1 (0.5)	0	1 (0.3)	3 (0.8)
Bacteriologic result								

	Incidence of TB							
	Low†		Intermediate‡		High§		All sites	
Characteristic	Non–MDR TB	MDR TB	Non–MDR TB	MDR TB	Non–MDR TB	MDR TB	Non–MDR TB	MDR TB
Smear +, culture +	65 (67.7)	64 (62.1)	51 (58.6)	39 (45.3)	145 (75.1)	142 (74.3)	261 (69.4)	245 (64.5)
Smear –, culture +	29 (30.2)	38 (36.9)	35 (40.2)	45 (52.3)	43 (22.3)	47 (24.6)	107 (28.5)	130 (34.2)
Unknown	0	1 (1.0)	0	0	0	0	0	1 (0.3)
Age group, y								
<18	3 (3.1)	3 (2.9)	0	0	0	6 (3.1)	3 (0.8)	9 (2.4)
18–24	9 (9.4)	17 (16.5)	8 (9.2)	14 (16.3)	22 (11.4)	25 (13.1)	39 (10.4)	56 (14.7)
25–44	47 (59.0)	68 (66.0)	32 (36.8)	34 (39.5)	91 (47.2)	91 (47.6)	170 (45.2)	193 (50.8)
45–64	24 (25.0)	12 (11.7)	39 (44.8)	28 (32.6)	66 (34.2)	62 (32.5)	129 (34.3)	102 (26.8)
≥65	12 (12.5)	3 (2.9)	3 (3.4)	2 (2.3)	11 (5.7)	2 (1.0)	26 (6.9)	7 (1.8)
Unknown	1 (1.0)	0	5 (5.7)	8 (9.3)	3 (1.6)	5 (2.6)	9 (2.4)	13 (3.4)
Currently employed								
Yes	33 (34.4)	46 (44.7)	34 (39.1)	35 (40.7)	77 (39.9)	63 (33.3)	144 (38.3)	144 (37.9)
Unknown	13 (13.5)	7 (6.8)	1 (1.1)	6 (7.0)	1 (1.0)	1 (0.5)	16 (4.3)	14 (3.7)
Work in a high-risk setting for TB								
Yes	3 (3.1)	8 (7.8)	4 (4.6)	6 (7.0)	11 (5.7)	9 (4.7)	18 (4.8)	23 (6.1)
Unknown	25 (26.0)	16 (15.5)	11 (12.6)	12 (14.0)	110 (57.0	107 56.0)	146 (38.8)	135 (35.5)
Excessive alcohol consumption								
Yes	21 (21.9)	11 (10.7)	33 (37.9)	20 (23.3)	54 (28.0)	48 (25.1)	108 (28.7)	79 (20.8)
Unknown	10 (10.4)	10 (9.7)	0	1 (1.2)	1 (0.5)	1 (0.5)	11 (2.9)	12 (3.2)
Imprisonment before current diagnosis								
Yes	2 (2.1)	4 (3.9)	9 (10.3)	8 (9.3)	4 (2.1)	18 (9.4)	15 (4.0)	30 (7.9)
Unknown	10 (10.4)	12 (11.7)	4 (4.6)	1 (1.2)	2 (1.0)	1 (0.5)	16 (4.3)	14 (3.7)
Current homelessness								
Yes	11 (11.5)	9 (8.7)	3 (3.4)	2 (2.3)	7 (3.6)	5 (2.6)	21 (5.6)	16 (4.2)
Unknown	2 (2.1)	2 (1.9)	4 (4.6)	2 (2.3)	3 (1.6)	1 (0.5)	9 (2.4)	5 (1.3)
Injectable drug use								
Yes	6 (6.3)	7 (6.8)	4 (4.6)	7 (8.1)	3 (1.6)	10 (5.2)	13 (3.5)	24 (6.3)
Unknown	9 (9.4)	12 (11.7)	3 (3.4)	2 (2.3)	19 (9.8)	15 (7.9)	31 (8.2)	29 (7.6)
Hepatitis B								
Yes	5 (5.2)	14 (13.6)	2 (2.3)	2 (2.3)	1 (0.5)	1 (0.5)	8 (2.1)	17 (4.5)
Unknown	18 (18.8)	20 (19.4)	11 (12.6)	5 (5.8)	21 (10.9)	78 (40.8)	50 (13.3)	103 (27.1)
Hepatitis C								
Yes	2 (2.1)	12 (11.7)	6 (6.9)	5 (5.8)	0	2 (1.0)	8 (2.1)	19 (5.0)
Unknown	20 (20.8)	22 (21.4)	31 (35.6)	22 (25.6)	22 (11.4)	81 (42.4)	73 (19.4)	125 (32.9)
Use of TNF inhibitor								
Yes	1 (1.0)	1 (1.0)	0	0	1 (0.5)	4 (2.1)	2 (0.5)	5 (1.3)
Unknown	8 (8.3)	7 (6.8)	4 (4.6)	5 (5.8)	39 (20.2)	42 (22.0)	51 (13.6)	54 (14.2)
Silicosis								
Yes	0	0	0	1 (1.2)	0	0	0	1 (0.3)
Unknown	0	3 (2.9)	2 (2.3)	1 (1.2)	3 (1.6)	1 (1.1)	5 (1.3)	5 (1.3)
Vaccination with *M. bovis* BCG								
Yes	38 (39.6)	42 (40.8)	67 (77.0)	72 (83.7)	160 (82.9)	184 (96.3)	267 (69.9)	300 (78.5)
Unknown	45 (46.9)	58 (56.3)	17 (19.5)	14 (16.3)	32 (16.6)	6 (3.1)	98 (25.7)	78 (20.4)

*Values are no. (%) or median (interquartile range). Unweighted analysis was used. MDR TB, multidrug-resistant tuberculosis; +, positive; –, negative; TNF, tumor necrosis factor.
†Austria, Belgium, Czech Republic, Denmark, Germany, Great Britain, Ireland, Netherlands, Italy, and Spain.
‡Belarus, Estonia, Latvia, and Portugal.
§Moldova and Romania.

Technical Appendix Table 3. Baseline drug resistance profiles for first-line and second-line drugs used for treatment of multidrug-resistant tuberculosis in TBNET study in Europe, 2010–2011*

Drug†	Incidence of TB in region, no. samples											
	Low‡				Intermediate§				High¶			
	Non–MDR TB, n = 96		MDR TB, n = 103		Non–MDR TB, n = 87		MDR TB, n = 86		Non–MDR TB, n = 193		MDR TB, n = 191	
	Tested	Resistant	Tested	Resistant	Tested	Resistant	Tested	Resistant	Tested	Resistant	Tested	Resistant
First line												
Pyrazinamide	92 (95.8)	0	97 (94.2)	52 (53.6)	44 (50.6)	1 (2.3)	70 (81.4)	49 (71.0)	1 (0.5)	0	10 (5.2)	4 (40.0)
Ethambutol	95 (99.0)	2 (2.1)	99 (96.1)	50 (50.5)	82 (94.3)	1 (1.2)	85 (98.9)	55 (64.7)	125 (67.8)	8 (6.4)	187 (97.9)	115 (61.5)
Streptomycin	65 (68.4)	3 (4.6)	93 (90.3)	78 (83.9)	82 (94.3)	11 (13.4)	85 (98.9)	82 (96.5)	125 (67.8)	22 (17.6)	187 (97.9)	171 (91.4)
≥1 non–first line	0	0	101 (97.1)	66 (65.4)	0	0	86 (100)	64 (74.4))	0	0	173 (86.4)	64 (37.0)
Class II												
Amikacin	0	0	95 (92.2)	17 (17.9)	0	0	85 (98.8)	25 (29.4)	0	0	1 (0.5)	0
Kanamycin	0	0	39 (37.9)	8 (20.5)	0	0	79 (91.9)	37 (46.8)	0	0	170 (89.0)	23 (13.5)
Capreomycin	0	0	88 (85.4)	15 (17.0)	0	0	84 (97.7)	26 (31.0)	0	0	94 (49.2)	4 (4.3)
≥1 second- line injectable	0	0	100 (97.1)	24 (24.0)	0	0	86 (100)	42 (48.8)	0	0	170 (89.0)	27 (15.9)
Class III												
Ofloxacin	0	0	69 (67.0)	16 (23.2)	0	0	86 (100)	26 (30.2)	0	0	169 (88.5)	14 (8.3)
Levofloxacin	0	0	16 (15.5)	1 (6.2)	0	0	7 (8.1)	1 (14.3)	0	0	10 (5.2)	2 (20.0)
Moxiflocacin	0	0	61 (59.2)	14 (23.0)	0	0	12 (14.0)	3 (25.0)	0	0	0	0
≥1 fluoroquinolone	0	0	96 (96.2)	21 (21.9)	0	0	86 (100)	26 (30.2)	0	0	170 (89.0)	15 (8.8)
Class IV												
ETO/PTO	0	0	98 (95.1)	47 (48.0)	0	0	86 (100)	36 (41.9)	0	0	170 (89.0)	36 (21.2)
PAS	0	0	54 (52.4)	10 (18.5)	0	0	68 (79.1)	10 (14.7)	0	0	175 (91.6)	2 (1.1)
DCS/TRD	0	0	53 (51.5)	6 (11.3)	0	0	69 (80.2)	13 (18.8)	0	0	100 (52.4)	5 (5.0)
Class V												
Linezolid	0	0	62 (60.2)	2 (3.2)	0	0	6 (7.0)	0	0		1 (0.5)	0
Imipenem	0	0	0	0	0	0	0	0	0	0	0	0
Meropenem	0	0	1 (1.0)	1 (100)	0	0	0	0	0	0	0	0
AMX/CVA	0	0	0	0	0	0	0	0	0	0	0	0
Clarithromycin	0	0	17 (16.5)	3 (17.7)	0	0	0	0	0	0	0	0

*Values are no. (%). Unweighted analysis was used. TB, tuberculosis; MDR TB, multidrug-resistant tuberculosis.
†ETO/PTO, ethionamide/prothionamide; PAS, para-aminosalicylic acid; DCS/TRD, cycloserine/terizidone; AMX/CVA, amoxicillin/clavulanic acid.
‡Austria, Belgium, Czech Republic, Denmark, Germany, Great Britain, Ireland, Netherlands, Italy, and Spain.
§Belarus, Estonia, Latvia, and Portugal.
¶Moldova and Romania.

Technical Appendix Table 4. Baseline drug resistance profiles for first-line and second-line drugs used for treatment of multidrug-resistant tuberculosis in TBNET study in Europe, 2010–2011*

	Incidence of TB in region, no. samples							
	Low, n = 37‡		Intermediate, n = 183§		High, n = 160¶		All MDR TB patients, n = 380	
Drug†	Tested	Resistant	Tested	Resistant	Tested	Resistant	Tested	Resistant
First line								
Pyrazinamide	35 (94.6)	17 (47.9) (29.3–66.5)	87 (47.5)	76 (88.0) (71.8–100)	9 (5.6)	4 (38.7) (0.0–100)	131 (34.4)	96 (73.9) (49.5–98.3)
Ethambutol	36(97.3)	18 (49.4) (37.7–61.2)	183 (100)	133 (72.6) (66.6–78.8)	157 (98.1)	97 (62.0) (11.0–100)	376 (98.9)	248 (66.0) (57.3–74.7)
Streptomycin	32 (86.5)	25 (76.9) (48.6–100)	183 (100)	182 (99.5) (96.9–100)	157 (98.1)	145 (92.4) (0.0–100)	372 (97.9)	352 (94.5) (86.9–100.0)
≥1 non–first line	36 (97.3)	23 (64.3) (38.1–90.5)	183 (100)	151 (82.2) (75.2–89.2)	144 (90.0)	54 (37.5) (0.0–92.7)	363 (95.5)	228 (62.8) (36.3–89.2)
Class II								
Amikacin	34 (91.9)	5 (15.4) (2.1–28.7)	178 (97.3)	48 (27.1) (23.3–31.0)	1 (0.6)	0	212 (55.8)	53 (25.2) (20.9–29.4)
Kanamycin	18 (48.6)	3 (17.2) (13.0–21.5)	176 (96.2)	97 (55.0) (48.1–61.9)	142 (88.8)	18 (12.3) (0.0–100)	336 (96.3)	118 (35.0) (4.5–65.5)
Capreomycin	30 (81.1)	4 (12.3) (0.6–23.9)	183 (100)	53 (29.2) (25.5–33.2)	85 (53.1)	3 (4.0) (0.0–24.0)	298 (78.4)	61 (20.3) (5.1–35.5)
≥1 second- line injectable	35 (94.6)	8 (22.8) (14.3–29.9)	183 (100)	99 (53.8) (48.0–59.5)	142 (88.8)	21 (14.8) (0.0–100)	360 (94.7)	127 (35.3) (9.8–60.9)
Class III								
Ofloxacin	26 (70.2)	5 (20.5) (13.9–27.1)	183 (100)	93 (50.8) (35.4–66.2)	141 (88.1)	11 (8.2) (0.0– 20.2)	350 (92.1)	110 (31.4) (2.5–60.4)
Levofloxacin	6 (16.2)	<1 (9.3) (2.7–15.9)	37 (20.2)	5 (14.3) NA)	8 (5.9)	2 (20.8) (0.0–100)	51 (13.4)	7 (14.7) (11.8–17.6)
Moxiflocacin	22 (59.5)	5 (20.7) (9.6–31.8)	9 (4.9)	1 (10.9) (0.0–100)	0	0	31 (8.2)	5 (17.9) (7.5– 28.3)
≥1 fluoroquinolone	34 (91.9)	7 (20.8) (13.0–28.7)	183 (100)	93 (50.8) (35.4–66.2)	142 (86.4)	12 (8.6) (0.0–29.7)	359 (94.5)	112 (31.3) (3.1–59.6)
Class IV								
ETO/PTO	35 (94.6)	18 (51.0) (22.1–79.9)	183 (100)	37 (20.1) (3.3–36.9)	142 (88.8)	32 (22.8) (0.0–100)	360 (94.7)	87 (24.2) (12.0–36.4)
PAS	17 (45.9)	2 (10.5) (0.0–26.7)	177 (96.7)	38 (21.6) (17.5–25.7)	144 (90.0)	2 (1.1) (0.0–4.5)	337 (88.7)	42 (12.3) (0.0–25.9)
DCS/TRD	17 (45.9)	3 (17.5) (0.0–44.8)	172 94.0)	69 (40.1) (26.5–53.7)	76 (47.5)	3 (4.2) (0.0–18.7)	265 (69.7)	75 (28.3) (5.3–51.4)
Class V								
Linezolid	18 (48.6)	<1	2 (0.1)	0	1 (0.6)	0	20 (5.2)	<1
Imipenem	0	0	0	0	0	0	0	0
Meropenem	<1	<1	0	0	0	0	<1	<1
AMX/CVA	0	0	0	0	0	0	0	0
Clarithromycin	12 (32.4)	2 (13.0) (8.8–17.2)	0	0	0	0	12 (3.2)	2 (13.0) (8.8– 17.2)

*Values are no. (%) and (95% CI). Weighted analysis was used. TB, tuberculosis; MDR TB, multidrug-resistant tuberculosis; NA, not applicable. Proportions may not reflect actual proportions because of use of a complex survey approach. Frequencies are rounded to nearest integer, except for frequency <1.
†inj, injectable; ETO/PTO, ethionamide/prothionamide; PAS, para-aminosalicylic acid; DCS/TRD, cycloserine/terizidone; AMX/CVA, amoxicillin/clavulanic acid.
‡Austria, Belgium, Czech Republic, Denmark, Germany, Great Britain, Ireland, Netherlands, Italy, and Spain.
§Belarus, Estonia, Latvia, and Portugal.
¶Moldova and Romania.

Technical Appendix Table 5. Risk factors for patients with multidrug-resistant tuberculosis analyzed by weighted analysis in TBNET study in Europe, 2010–2011*

Factor	Non–MDR TB, n = 376	MDR TB, n = 380	Univariable analysis		Multivariable analysis (parsimonious)	
			OR (95% CI)	p value	OR (95% CI)	p value
Sex						
F	111	141	1	NA	1	NA
M	265	239	0.70 (0.50–0.99)	0.043	0.67 (0.48–0.96)	0.029
Age group, y						
<45	212	258	1.58 (1.13–2.20)	0.011	1.66 (1.01–2.52)	0.020
≥45	155	109	1		1	
Unknown or missing	4	10	NA	NA	NA	NA
Body mass index						
<18	31	48	2.03 (1.17–3.52)	0.015	NA	NA
18–<25	276	261	1		NA	NA
≥25	38	49	1.41 (0.67–3.00)	0.414	NA	NA
Currently employed						
Yes	144	144	1		NA	NA
No	211	222	1.64 (0.56–4.80)	0.339	NA	NA
Unknown or missing	16	14			NA	NA
Foreign born						
Yes	63	94	0.55 (0.22–1.40)	0.187	NA	NA
No	313	286	1	NA	NA	NA
Imprisonment before current diagnosis						
Yes	15	30	2.32 (0.92–5.86)	0.073	NA	NA
No	345	336	1	NA	NA	NA
Unknown or missing	16	14	NA	NA	NA	NA
Current homelessness						
Yes	21	16	0.32 (0.10–1.01)	0.052	4.22 (1.07–16.65)	0.041
No	346	359	1	NA	1	NA
Unknown or missing	9	5	NA	NA	NA	NA
Injectable drug user						
Yes	13	24	0.91 (0.18–4.49)	0.897	NA	NA
No	332	327	1	NA	NA	NA
Unknown or missing	31	29	NA	NA	NA	NA
HIV infected						
Yes	16	25	0.77 (0.23–2.62)	0.661	NA	NA
No	320	345	1	NA	NA	NA
Not tested	36	9	NA	NA	NA	NA
Missing	4	1	NA	NA	NA	NA
Diabetes						
Yes	20	16	0.78 (0.30–2.04)	0.588	NA	NA
No	354	356	1	NA	NA	NA
Unknown or missing	2	8	NA	NA	NA	NA
Previous TB treatment						
Yes	33	133	8.171 (5.58–12.20)	<0.001	9.29 (6.11–14.11)	<0.001
No	339	244	1	NA	1	NA
Unknown or missing	4	3	NA	NA	NA	NA

*Proportions might not reflect actual proportions because of use of a complex survey approach. MDR TB, multidrug-resistant tuberculosis; OR, odds ratio, NA, not applicable.

Reference

1. European Centre for Disease Prevention and Control/WHO Regional Office for Europe. Tuberculosis surveillance and monitoring in Europe 2013. Stockholm: The Centre; 2013.
